# Gene Expression Analysis of Papillary Thyroid Carcinoma With Lymph Node Metastasis and Radioiodine Refractivity

**DOI:** 10.7759/cureus.87001

**Published:** 2025-06-29

**Authors:** Nur Fadhlina Mohamad Pakarulrazy, Nadiah Abu, Shahrun Niza Abdullah Suhaimi, Nadzlee Harith Paisol, Reena Rahayu Md Zin, Nani H MdLatar, Nurul Syakima Ab Mutalib

**Affiliations:** 1 UKM Medical Molecular Biology Institute, Universiti Kebangsaan Malaysia, Kuala Lumpur, MYS; 2 Department of Surgery, Faculty of Medicine, Universiti Kebangsaan Malaysia Medical Centre, Kuala Lumpur, MYS; 3 Department of Surgery, Universiti Putra Malaysia, Serdang, MYS; 4 Department of Pathology, Faculty of Medicine, Universiti Kebangsaan Malaysia Medical Centre, Kuala Lumpur, MYS; 5 Department of Surgery, Sunway Medical Centre, Petaling Jaya, MYS

**Keywords:** cancer biomarker, microarray, papillary thyroid carcinoma, precision medicine, radioiodine, refractory

## Abstract

Background/aim: Papillary thyroid carcinoma (PTC) is the most prevalent form of thyroid cancer (TC) and is generally associated with a favorable prognosis. Nevertheless, aggressive variants of PTC that exhibit metastasis and resistance to radioiodine (RAI) therapy present significant clinical challenges. This study sought to generate a preliminary dataset on gene expression in RAI-refractory PTC using microarray analysis.

Materials and methods: Fresh frozen thyroid tissues were collected from PTC patients without lymph node metastasis and RAI avidity (n = 5), PTC patients with lymph node metastasis and RAI refractoriness (n = 5), and adjacent normal thyroid tissues (n = 4). The samples were cryosectioned, stained with hematoxylin and eosin, and confirmed by a pathologist. Nucleic acids were extracted using the AllPrep DNA/RNA/miRNA Universal Kit (Qiagen, Germany), and RNA quantity, purity, and integrity were assessed. RNA samples were amplified, labelled using the Agilent Low Input Quick Amp Labeling Kit (Agilent Technologies, Santa Clara, CA, US), and purified using the RNeasy Mini Kit (Qiagen). Cy3-labelled cRNA was fragmented and hybridized to Agilent SurePrint G3 Human GE v3 8 × 60 K microarray slides. Data were analyzed using AltAnalyze software and the iDEP web application.

Results: Our results revealed distinct expression patterns between RAI-avid and RAI-refractory PTC, with significant downregulation observed in key thyroid hormone synthesis genes, such as *TPO*, *DIO1*, and *SLC26A4*, across both groups. Notably, *TG* showed a variable expression pattern, suggesting its complex role in PTC pathophysiology. Pathway analysis highlighted the disruption of metabolic and immune-related pathways, emphasizing the altered physiological state of RAI-refractory PTC.

Conclusion: This study provides essential insights into the molecular underpinnings of RAI resistance in PTC and offers a foundation for future research aimed at developing targeted therapies that could enhance treatment efficacy and patient outcomes.

## Introduction

Thyroid cancer is the most common endocrine malignancy, with rising global incidence particularly among women in high- and middle-income countries [1]. In 2022, GLOBOCAN reported approximately 586,000 new cases and 43,000 deaths worldwide, with Asia contributing over half the burden [2]. In Malaysia, newly diagnosed thyroid cancer cases rose from 4,829 in 2010 to 6,583 in 2019, alongside a 14% increase in age-standardized death rates, underscoring its growing health and socioeconomic impact [1].

Among thyroid malignancies, papillary thyroid carcinoma (PTC) accounts for over 90% of differentiated thyroid carcinomas and typically carries a favorable prognosis, with reported 10-year survival rates exceeding 90% [3]. However, a subset of PTCs displays aggressive behavior, including extrathyroidal extension, lymph node metastasis (LNM), and distant metastasis, all of which are associated with a higher risk of recurrence and mortality [4-7].

Radioactive iodine (RAI) ablation is a key postoperative therapy for intermediate- and high-risk PTC, functioning via the sodium/iodide symporter (NIS) [8]. However, up to 60% of metastatic PTCs become RAI-refractory (RAI-R), often due to dedifferentiation and loss of iodine uptake [9]. RAI-R is indicated by absent or incomplete RAI uptake on scans, disease progression despite RAI uptake, or lack of response after high cumulative RAI doses [8,10]. These criteria require careful interpretation within the clinical context.

RAI-R has been linked to loss of differentiation, often mediated by the downregulation of genes such as SLC5A5, TPO, TSHR, and TG, which are essential for iodide metabolism and thyroid hormone synthesis [11]. This dedifferentiation is frequently associated with oncogenic alterations, including BRAFV600E and TERT promoter mutations, which can impair NIS expression through MAPK pathway activation and epigenetic silencing [12]. LNM, particularly in the lateral neck, further correlates with reduced RAI uptake and treatment resistance [5].

Despite growing interest globally, few studies have explored the transcriptomic landscape of RAI-R PTC in Southeast Asia [13]. Given the potential impact of ethnicity and environmental exposures on tumor biology, this study aimed to compare gene expression profiles between RAI-avid (RAI-A) and RAI-R PTC in Malaysian patients using microarray analysis. To our knowledge, this represents the first transcriptomic investigation of RAI-R in this population, with the goal of uncovering context-specific molecular signatures and informing future therapeutic strategies.

## Materials and methods

Clinical specimens

Fresh frozen thyroid tumor tissues were collected from 10 patients diagnosed with PTC who underwent surgery at Hospital Canselor Tuanku Muhriz UKM (HCTM), Kuala Lumpur, between 2011 and 2020. Five tumor samples were obtained from patients without LNM, and five from patients with LNM. Additionally, four adjacent normal thyroid tissues were collected from PTC patients during surgery.

Clinical records were retrospectively reviewed. Inclusion criteria included a confirmed histopathological diagnosis of PTC, receipt of at least one RAI treatment, and availability of follow-up data for a minimum of two years following total thyroidectomy. During follow-up, patients were monitored with serum thyroid-stimulating hormone (TSH), thyroglobulin (Tg), anti-Tg antibodies, and free T4. Imaging surveillance included neck ultrasound, chest radiography, 131I whole-body scans, and fluorodeoxyglucose (FDG)-PET/CT scans for detecting metabolically active or RAI-R disease, when clinically indicated.

RAI-R disease was defined according to the 2015 American Thyroid Association (ATA) guidelines and international consensus criteria [8,10]. A patient was classified as RAI-R if they met one or more of the following: (i) no RAI uptake in metastatic lesions on a diagnostic or post-therapy scan, (ii) progressive disease despite RAI uptake, (iii) mixed RAI uptake with some lesions showing no activity, or (iv) disease progression after a cumulative RAI activity exceeding 600 mCi. Based on this classification, patients without LNM and RAI-avid were assigned to the RAI-A group, and those with LNM and RAI refractoriness were classified as RAI-R. Clinical outcomes were further categorized as either remission or persistent disease, according to the same ATA guidelines [10].

This study was approved by the Universiti Kebangsaan Malaysia Research Ethics Committee (UKM PPI/111/8/JEP-2020-677), and written informed consent was obtained from all participants.

Cryosectioning and hematoxylin and eosin staining

Tumor and adjacent normal thyroid tissues were dissected, snap-frozen in liquid nitrogen, and stored until processing. All samples were cryosectioned at a thickness of 5 µm and stained with hematoxylin and eosin. The percentages of tumor cells and normal cell contents were confirmed by a pathologist. Only tumor samples containing at least 80% cancerous cells and normal adjacent thyroid tissues with less than 20% necrosis were selected for microarray analysis to ensure sample purity and RNA integrity.

Total RNA extraction

Total RNA was extracted from frozen thyroid tissue using the AllPrep DNA/RNA/miRNA Universal Kit (Qiagen, Germany). RNA quantity and purity were assessed with a NanoDrop 2000c spectrophotometer (Thermo Fisher Scientific, Waltham, MA, US), and only samples with 260/280 absorbance ratios between 1.8 and 2.0 were included. RNA integrity was evaluated using the Agilent Bioanalyzer 2100 (Agilent Technologies, Santa Clara, CA, US), and samples with an RNA Integrity Number (RIN) greater than 5 were accepted. Although a RIN of 7 or higher is typically preferred for microarray studies, a lower threshold was used due to the limited availability of high-quality archived tissue and the retrospective study design. To minimize technical variability, all samples were processed using standardized extraction and hybridization protocols.

Microarray hybridization

Total RNA concentrations were normalized to 100 ng prior to labeling. Amplification and Cy3 labeling of RNA were performed using the Agilent Low Input Quick Amp Labeling Kit, One-Color. Cy3-labelled complementary RNA (cRNA) was purified using the RNeasy Mini Kit (Qiagen), and dye incorporation and cRNA yield were assessed using the NanoVue Plus Spectrophotometer (GE Healthcare, UK).

A total of 0.825 μg of Cy3-labelled cRNA with a specific activity greater than 6 pmol Cy3/μg cRNA was fragmented at 60°C for 30 minutes in a 25 μL reaction volume containing 25X Agilent fragmentation buffer and 10X blocking agent. The fragmented cRNA was then hybridized onto the Agilent SurePrint G3 Human Gene Expression v3 8 × 60 K Microarray (Design ID: 072363) at 65°C for 17 hours in a rotating hybridization oven.

Post-hybridization washing was carried out using the Agilent Gene Expression Wash Buffer Kit. Microarray slides were scanned immediately using an Agilent SureScan Microarray Scanner (G4900DA) at 3 μm resolution, 532 nm wavelength (Cy3), and an extended dynamic range of 10%-100%. Signal intensities were extracted using Agilent Feature Extraction Software (protocol GE1_1200_Jun14), and raw data were saved in .txt format for further analysis.

The Agilent SurePrint G3 Human Gene Expression v3 8 × 60 K Microarray contains 26,803 unique Entrez protein-coding genes and 30,606 unique long non-coding RNAs, enabling comprehensive transcriptome profiling.

Data preprocessing

Raw Agilent Feature Extraction text files were processed using AltAnalyze (version 2.1.4) [14] with the Process Feature Extraction Files workflow. The dataset was based on the Agilent expression array platform for *Homo sapiens*, using the EnsMart72 gene database. Expression values were extracted from the green channel and imported in log2 format. Data were quantile normalized to reduce inter-array variability, and all sample values were retained in the final output matrix. Batch correction was not applied as only two Agilent microarray chips were used, each containing a mix of experimental conditions. This distribution reduced the risk of batch effects [15]. The processed data were used for expression analysis, as described below.

Identification of differentially expressed genes

The normalized expression data were analyzed using integrated Differential Expression & Pathway analysis (iDEP) v2.0 [16,17] with default settings. The limma statistical method [18] in iDEP was used to identify differentially expressed genes (DEGs). Genes with a false discovery rate (FDR) of less than 0.05 and a minimal log2 fold change (log2FC) equal to or greater than one were considered significantly differentially expressed [19].

Pathway enrichment analysis

Pathway enrichment analysis of DEGs was performed using iDEP v2.0, based on the Kyoto Encyclopedia of Genes and Genomes (KEGG) database [20]. Gene set enrichment analysis (GSEA) was conducted in the preranked mode using the fgsea algorithm [21,22]. Pathways with an FDR less than 0.05 were considered significantly enriched.

Data and code availability

The microarray data have been deposited in the National Center for Biotechnology Information (NCBI) Gene Expression Omnibus (GEO) under accession number GSE299988. All analyses were performed using publicly available, graphical user interface (GUI)-based software tools (AltAnalyze v2.1.4 and iDEP v2.0) with default settings unless otherwise specified. No custom scripts or code were generated.

## Results

Clinicopathological data

A total of 10 PTC patients were analyzed, equally divided between the RAI-A (n = 5) and RAI-R (n = 5) groups. Most patients in both groups were Malay, with two Chinese patients in the RAI-R group. All RAI-A patients were female, whereas the RAI-R group included three women and two men. The mean age at diagnosis was higher in the RAI-R group (61.20 ± 15.66 years) compared to the RAI-A group (51.40 ± 15.25 years), with a similar distribution of patients above and below 55 years.

Clinically, most RAI-A patients were Stage I and classified as low to intermediate ATA risk. In contrast, RAI-R patients showed a shift toward advanced stages (Stage IV in three cases) and high-risk ATA classification. The RAI-R group also exhibited more aggressive features, including T4 tumors, tall cell variant histology, multifocality, bilaterality, and calcification. Tumors measuring 4 cm or larger were present in both groups, with slightly more cases in RAI-A.

LNM were absent in all RAI-A cases but present in all RAI-R patients, with most having more than five metastatic nodes and an LNM ratio exceeding 0.3. Distant metastases were identified in three RAI-R patients.

All RAI-A patients underwent total thyroidectomy with central neck dissection, received cumulative RAI doses less than 600 mCi, and achieved remission. In contrast, RAI-R patients more often required both lateral and central neck dissection but remained refractory despite receiving comparable RAI doses. All RAI-R cases were FDG-avid and failed to achieve remission. Among them, four patients experienced a late loss of RAI uptake after initial avidity, while one exhibited mixed RAI uptake across lesions, reflecting heterogeneous or progressive loss of RAI-A. Clinicopathological data for the 10 PTC patients included in the discovery phase using microarray analysis are summarized in Table 1. Detailed characteristics of the RAI-A and RAI-R subgroups are provided in Tables 2, 3, respectively.

**Table 1:** Summary of clinicopathological characteristics in RAI-A and RAI-R patients. *The tumor initially exhibited RAI uptake but subsequently lost this ability. ^#^RAI uptake was present in neck tissue but absent in lung nodules. PTC: papillary thyroid carcinoma; RAI: radioactive iodine; LNM: lymph node metastasis; AJCC: American Joint Committee on Cancer; ATA: American Thyroid Association; FDG: fluorodeoxyglucose; RAI-R: radioactive iodine-refractory; RAI-A: radioactive iodine-avid.

Characteristics of PTC patients		RAI-A (n = 5)	RAI-R (n = 5)
Demographics	Gender		
	Female/male	5/0	3/2
	Race		
	Malay/Chinese	4/1	3/2
	Age at diagnosis (years)		
	<55/≥55	2/3	2/3
	Mean ± SD	51.40 ± 15.25	61.20 ± 15.66
Staging & risk stratification	AJCC stage		
	I/II/III/IV	4/1/0/0	1/1/0/3
	ATA risk stratification system		
	Low/intermediate/high	2/3/0	0/2/3
Tumor characteristics	Primary tumor, T		
	T1/T2/T3/T4	0/2/3/0	1/1/1/2
	Histological variant		
	Classic/cribriform morular/tall cell	4/1/0	4/0/1
	Tumor size (cm)		
	<4/≥4	2/3	3/2
	Tumor focality		
	Unifocal/multifocal	5/0	2/3
	Tumor laterality		
	Unilateral/bilateral	5/0	2/3
	Calcification		
	No/yes	5/0	1/4
	Extrathyroidal extension		
	No/yes	4/1	3/2
	Vascular invasion		
	No/yes	2/3	3/2
Nodal & metastatic status	Regional LNM, N		
	N0/N1	5/0	0/5
	Lateral LNM		
	No/yes	-	1/4
	No. of LNM		
	<5/≥5	-	2/3
	LNM ratio		
	≤0.3/>0.3	-	1/4
	Distant metastasis, M		
	M0/M1	5/0	2/3
Treatment & response	Surgical type		
	Total/completion thyroidectomy	5/0	4/1
	LN dissection		
	Central only/lateral & central	3/2	1/4
	RAI response		
	Avid/refractive	5/0	0/5
Imaging & biological behavior	RAI-refractory type		
	Late loss of RAI uptake/mixed RAI uptake	-	4*/1^#^
	FDG-avid		
	No/yes	-	0/5
	Remission		
	No/yes	0/5	5/0

**Table 2:** Detailed clinicopathological characteristics of RAI-A patients. PTC: papillary thyroid carcinoma; RAI: radioactive iodine; RAI-A: radioactive iodine-avid; LNM: lymph node metastasis; AJCC: American Joint Committee on Cancer; ATA: American Thyroid Association; FDG: fluorodeoxyglucose.

Characteristics of patients		RAI-A patients				
		RAIA1	RAIA2	RAIA3	RAIA4	RAIA5
Demographics	Gender	Female	Female	Female	Female	Female
	Race	Chinese	Malay	Malay	Malay	Malay
	Age at diagnosis (years)	59	61	69	41	27
Staging & risk stratification	AJCC stage	I	I	II	I	I
	ATA risk stratification system	Low	Low	Intermediate	Intermediate	Intermediate
Tumor characteristics	Primary tumor (T)	T2	T2	T3a	T3b	T3a
	Histological variant	Classic	Classic	Cribriform morular	Classic	Classic
	Tumor size (mm)	22	30	80	40	55
	Tumor focality	Unifocal	Unifocal	Unifocal	Unifocal	Unifocal
	Tumor laterality	Unilateral	Unilateral	Unilateral	Unilateral	Unilateral
	Calcification	No	No	No	No	No
	Extrathyroidal extension	No	No	No	Yes	No
	Vascular invasion	No	No	Yes	Yes	Yes
Nodal & metastatic status	Regional LNM (N)	N0	N0	N0	N0	N0
	Lateral LNM	-	-	-	-	-
	No. of LNM	-	-	-	-	-
	LNM ratio	-	-	-	-	-
	Distant metastasis (M)	M0	M0	M0	M0	M0
Treatment & response	Surgical type	Total thyroidectomy	Total thyroidectomy	Total thyroidectomy	Total thyroidectomy	Total thyroidectomy
	LN dissection	Central node	Central node	Lateral & central node	Central node	Lateral & central node
	RAI response	Avid	Avid	Avid	Avid	Avid
Imaging & biological behavior	RAI-refractory type	-	-	-	-	-
	FDG-avid	-	-	-	-	-
	Remission	Yes	Yes	Yes	Yes	Yes

**Table 3:** Detailed clinicopathological characteristics of RAI-R patients. PTC: papillary thyroid carcinoma; RAI: radioactive iodine; RAI-R: radioactive iodine-refractory; LNM: lymph node metastasis; AJCC: American Joint Committee on Cancer; ATA: American Thyroid Association; FDG: fluorodeoxyglucose.

Characteristics of patients		RAI-R patients				
		RAIR1	RAIR2	RAIR3	RAIR4	RAIR5
Demographics	Gender	Female	Female	Male	Male	Female
	Race	Chinese	Chinese	Malay	Malay	Malay
	Age at diagnosis (years)	79	54	74	64	35
Staging & risk stratification	AJCC stage	II	IVB	IVB	IVB	I
	ATA risk stratification system	Intermediate	High	High	High	Intermediate
Tumor characteristics	Primary tumor (T)	T2	T3a	T4a	T4a	T1b
	Histological variant	Tall cell	Classic	Classic	Classic	Classic
	Tumor size (mm)	35	42	55	25	12
	Tumor focality	Unifocal	Unifocal	Multifocal	Multifocal	Multifocal
	Tumor laterality	Unilateral	Unilateral	Bilateral	Bilateral	Bilateral
	Calcification	No	Yes	Yes	Yes	Yes
	Extrathyroidal extension	No	No	Yes	Yes	No
	Vascular invasion	No	No	Yes	Yes	No
Nodal & metastatic status	Regional LNM (N)	N1a	N1b	N1b	N1b	N1b
	Lateral LNM	No	Yes	Yes	Yes	Yes
	No. of LNM	3	8	7	3	25
	LNM ratio	0.43	0.89	0.14	0.33	0.49
	Distant metastasis (M)	M0	M1	M1	M1	M0
Treatment & response	Surgical type	Total thyroidectomy	Total thyroidectomy	Total thyroidectomy	Total thyroidectomy	Completion thyroidectomy
	LN dissection	Central node	Lateral & central node	Lateral & central node	Lateral & central node	Lateral & central node
	RAI response	Refractive	Refractive	Refractive	Refractive	Refractive
Imaging & biological behavior	RAI-refractory type	Tumor tissue that no longer takes up RAI despite previous uptake	Tumor tissue that no longer takes up RAI despite previous uptake	RAI uptake in some lesions only	Tumor tissue that no longer takes up RAI despite previous uptake	Tumor tissue that no longer takes up RAI despite previous uptake
	FDG-avid	Yes	Yes	Yes	Yes	Yes
	Remission	No	No	No	No	No

Preprocessing and normalization of microarray data

The microarray analysis generated raw signal intensities from 62,973 probes, comprising 26,803 unique Entrez gene probes, 30,606 unique long non-coding RNA probes, and 3,000 replicated biological probes. Raw signal values were log₂-transformed and normalized using the quantile normalization method. After filtering out low-quality probes, a total of 58,342 probes were retained for each sample. The lowest probe intensity for each sample was 2.73, and the highest probe intensity was 17.91. The average probe intensity for each sample is 4.65. Several genes are represented by unique probes.

Distinct gene expression profiles in RAI-A and RAI-R PTC

**Figure 1:**
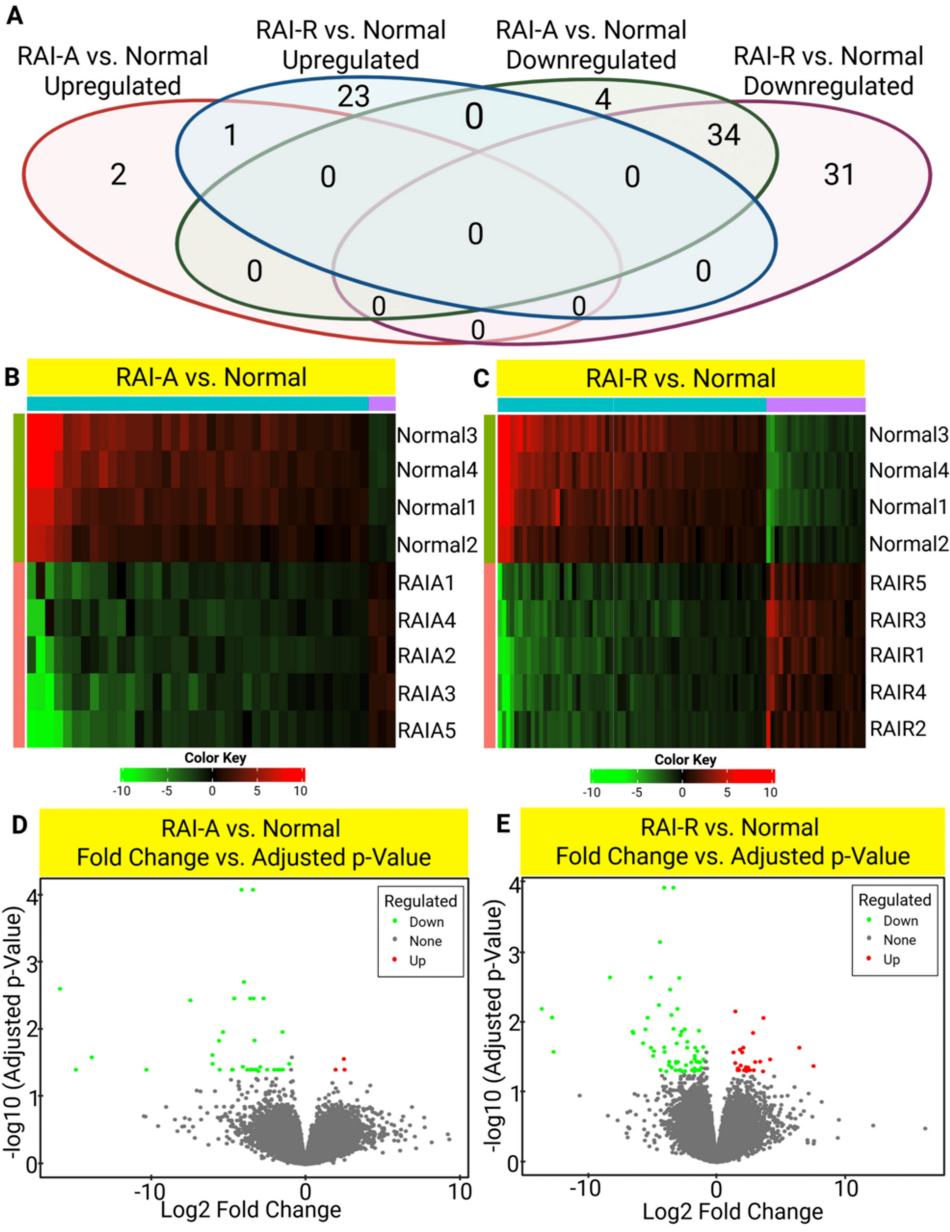
Differential gene expression profiles in radioactive iodine-avid (RAI-A) and radioactive iodine-refractory (RAI-R) papillary thyroid carcinoma (PTC). Differential gene expression profiles in RAI-A and RAI-R PTC. (A) Venn diagram showing the number of DEGs between adjacent normal thyroid tissues and PTC tissues with RAI-A or RAI-R. DEGs were identified using the limma statistical method in iDEP v2.0, with a false discovery rate < 0.05 and a fold change ≥ 2. (B) Heatmap of the top 100 upregulated and downregulated DEGs in RAI-A compared to adjacent normal thyroid tissues. (C) Heatmap of the top 100 upregulated and downregulated DEGs in RAI-R compared to adjacent normal thyroid tissue. The color scale represents the log2 fold change, with red indicating upregulated DEGs and green indicating downregulated DEGs. (D, E) Volcano plots showing the distribution of DEGs between RAI-A (D) and RAI-R (E) compared with adjacent normal thyroid tissues. Significantly upregulated DEGs are highlighted in red, whereas those significantly downregulated DEGs are marked in green. DEGs: differentially expressed genes.

Differential gene expression analysis revealed distinct gene expression profiles between PTC tissues with RAI-A and RAI-R compared to normal thyroid tissues. As shown in the Venn diagram (Figure 1A), two genes were uniquely upregulated in RAI-A, whereas 23 genes were upregulated in RAI-R compared to normal thyroid tissue. Similarly, four genes were uniquely downregulated in RAI-A, whereas 31 genes were downregulated in RAI-R. No overlap was observed between the upregulated or downregulated gene sets of RAI-A and RAI-R, suggesting distinct transcriptional profiles associated with their respective RAI responses.

Heatmaps of the top 100 DEGs (Figures 1B, 1C) demonstrate a clear separation between normal, RAI-A, and RAI-R samples, further supporting their distinct gene expression patterns. Volcano plots (Figures 1D, 1E) highlight significantly upregulated (red) and downregulated (green) genes in RAI-A and RAI-R, respectively, based on thresholds of FDR < 0.05 and a fold change ≥ 2. Notably, RAI-R samples exhibited a larger number of downregulated genes, suggesting more extensive transcriptional repression associated with RAI-R. Moreover, both groups showed significant changes in gene expression compared to normal thyroid tissues, reflecting their different biological responses to RAI treatment.

To minimize probe-specific variability, expression values from multiple significant probes targeting the same gene (e.g., TFF3, represented by probes A_23_P393099 and A_33_P3334305) were averaged prior to downstream analysis. This approach ensured more accurate estimation of gene-level expression changes.

Differential gene expression analysis of RAI-A, RAI-R, and normal thyroid tissues identified several significant DEGs in each group. Table 4 summarizes the top 10 most significant DEGs in RAI-A and RAI-R based on their log2FC and adjusted p-values (AdjPval). For instance, TFF3 and LRP1B were significantly downregulated in both groups, with a more pronounced decrease in RAI-R. In contrast, genes such as TPO showed more substantial and significant downregulation in RAI-A, reflecting unique transcriptional changes associated with RAI-A.

**Table 4:** Top 10 significant differentially expressed genes in RAI-A and RAI-R. Log2FC: log2 fold change; AdjPval: adjusted p-value; ns: not significant; RAI-A: radioactive iodine-avid; RAI-R: radioactive iodine-refractory.

Gene symbol/Ensembl ID	RAI-A vs normal		RAI-R vs normal		RAI-R vs RAI-A	
	Log2FC	AdjPval	Log2FC	AdjPval	Log2FC	AdjPval
DIO1	-3.39	9.18E-05	-3.37	1.01E-04	0.01	ns
LINGO2	-4.12	9.18E-05	-4.09	1.01E-04	0.03	ns
LOC401127	-3.97	2.08E-03	-4.42	6.25E-04	-0.44	ns
TPO	-15.84	2.63E-03	-13.62	5.87E-03	2.22	ns
ENSG00000288520	-3.57	3.64E-03	-3.63	3.06E-03	-0.07	ns
STAB2	-3.35	3.64E-03	-3.06	5.87E-03	0.28	ns
TFF2	-2.71	3.64E-03	-2.92	2.08E-03	-0.22	ns
LRP1B	-4.60	3.64E-03	-5.13	2.04E-03	-0.53	ns
TFF3	-8.85	2.22E-02	-10.56	4.93E-03	-1.71	ns
CUX2	-1.48	1.13E-02	-1.39	1.22E-02	0.10	ns

Additionally, unique sets of DEGs were identified in RAI-A and RAI-R, with no overlap between the two groups. Table 5 highlights the unique DEGs in RAI-R, which include genes such as ANTXR2 and SH2D6, both of which are strongly associated with RAI-R. The larger number of unique DEGs in RAI-R suggests more profound transcriptional dysregulation in this group, potentially contributing to RAI resistance. The absence of shared DEGs between RAI-A and RAI-R further reinforces the idea that these two PTC subtypes have distinct molecular profiles, each reflecting a different biological response to RAI. The unique RAI-R DEGs identified in Table 5 may represent potential therapeutic targets for overcoming RAI-R in PTC. A full list of significant DEGs can be found in Tables 6, 7.

**Table 5:** Unique differentially expressed genes in RAI-R (highlighting genes specifically dysregulated in RAI-R cases). Log2FC: log2 fold change; AdjPval: adjusted p-value; ns: not significant; RAI-A: radioactive iodine-avid; RAI-R: radioactive iodine-refractory.

Gene symbol/Ensembl ID	RAI-A vs normal		RAI-R vs normal		RAI-R vs RAI-A	
	Log2FC	AdjPval	Log2FC	AdjPval	Log2FC	AdjPval
ANTXR2	0.87	ns	1.44	6.39E-03	0.57	ns
EDIL3	2.25	ns	3.63	7.96E-03	1.38	ns
PKHD1L1	-2.13	ns	-2.74	1.26E-02	-0.61	ns
ESPN	0.51	ns	2.82	1.31E-02	2.31	ns
C9orf92	-2.20	ns	-2.82	1.42E-02	-0.63	ns
KIF19	-4.81	ns	-5.73	1.86E-02	-0.92	ns
ASPG	-2.36	ns	-3.12	1.87E-02	-0.77	ns
SH2D6	-2.88	ns	-4.09	2.13E-02	-1.21	ns
KIAA0408	4.29	ns	6.42	2.15E-02	2.13	ns
REV1	1.74	ns	2.06	2.15E-02	0.33	ns

**Table 6:** Summary of significant differentially expressed genes (overall significant genes in RAI-A and RAI-R). Log2FC: log2 fold change; AdjPval: adjusted p-value; ns: not significant; RAI-A: radioactive iodine-avid; RAI-R: radioactive iodine-refractory.

Gene symbol/Ensembl ID	RAI-A vs normal		RAI-R vs normal		RAI-R vs RAI-A	
	Log2FC	AdjPval	Log2FC	AdjPval	Log2FC	AdjPval
DIO1	-3.39	9.18E-05	-3.37	1.01E-04	0.01	ns
LINGO2	-4.12	9.18E-05	-4.09	1.01E-04	0.03	ns
LOC401127	-3.97	2.08E-03	-4.42	6.25E-04	-0.44	ns
TPO	-15.84	2.63E-03	-13.62	5.87E-03	2.22	ns
ENSG00000288520	-3.57	3.64E-03	-3.63	3.06E-03	-0.07	ns
LRP1B	-4.60	3.64E-03	-5.13	2.04E-03	-0.53	ns
STAB2	-3.35	3.64E-03	-3.06	5.87E-03	0.28	ns
TFF2	-2.71	3.64E-03	-2.92	2.08E-03	-0.22	ns
CUX2	-1.48	1.13E-02	-1.39	1.22E-02	0.10	ns
SEMA3D	-5.32	1.13E-02	-5.39	7.88E-03	-0.06	ns
PPARGC1A	-3.29	1.51E-02	-3.53	7.21E-03	-0.24	ns
GRIN2C	-5.58	1.52E-02	-5.55	1.17E-02	0.03	ns
TFF3	-8.85	2.22E-02	-10.56	4.93E-03	-1.71	ns
CDH16	-6.01	2.47E-02	-4.07	ns	1.94	ns
CRABP1	-13.79	2.67E-02	-12.70	2.48E-02	1.10	ns
ENSG00000256484	-0.88	2.67E-02	-0.81	2.52E-02	0.08	ns
PCGF3	2.48	2.82E-02	1.53	ns	-0.95	ns
lnc-CDK9-1	-1.05	3.33E-02	-1.06	2.11E-02	-0.01	ns
MT1G	-6.00	3.33E-02	-6.55	1.26E-02	-0.55	ns
IMPA2	-4.04	3.67E-02	-3.70	3.45E-02	0.34	ns
ENSG00000260403	-2.92	3.71E-02	-3.37	1.14E-02	-0.44	ns
ADAM33	-3.45	4.06E-02	-3.15	3.61E-02	0.30	ns
CCL21	-14.81	4.06E-02	-10.67	ns	4.15	ns
ENSG00000283766	-3.76	4.06E-02	-3.51	4.19E-02	0.25	ns
ENSG00000285569	-1.46	4.06E-02	-1.51	2.41E-02	-0.06	ns
ENSG00000287198	-1.72	4.06E-02	-1.72	3.13E-02	0.00	ns
EYA2	-3.56	4.06E-02	-3.20	4.58E-02	0.36	ns
GRIN3B	1.94	4.06E-02	1.09	ns	-0.85	ns
HGD	-4.74	4.06E-02	-4.37	4.57E-02	0.36	ns
HOXC-AS2	-0.93	4.06E-02	-0.91	3.40E-02	0.02	ns
HS6ST3	-2.47	4.06E-02	-2.09	ns	0.39	ns
KLF13	-2.01	4.06E-02	-2.29	1.77E-02	-0.28	ns
LGI3	-3.24	4.06E-02	-2.80	ns	0.44	ns
LINC00092	-3.18	4.06E-02	-2.84	4.57E-02	0.34	ns
lnc-SPATA6L-1	-1.81	4.06E-02	-1.66	4.57E-02	0.15	ns
MPIG6B	-2.02	4.06E-02	-2.49	1.17E-02	-0.47	ns
MT1H	-5.53	4.06E-02	-6.49	1.32E-02	-0.95	ns
OGDHL	-3.23	4.06E-02	-4.50	5.16E-03	-1.26	ns
PARD3-DT	2.52	4.06E-02	2.27	4.15E-02	-0.25	ns
PLA2R1	-4.69	4.06E-02	-5.12	2.15E-02	-0.43	ns
SLC5A8	-1.56	4.06E-02	-1.68	2.15E-02	-0.12	ns
CFD	-3.04	4.25E-02	-3.02	3.48E-02	0.02	ns
ANTXR2	0.87	ns	1.44	6.39E-03	0.57	ns
ASPG	-2.36	ns	-3.12	1.87E-02	-0.77	ns
ATP5MC3	-0.77	ns	-1.32	4.57E-02	-0.54	ns
ATP6AP1L	1.50	ns	1.63	4.58E-02	0.12	ns
C9orf92	-2.20	ns	-2.82	1.42E-02	-0.63	ns
CA4	-3.74	ns	-3.93	4.77E-02	-0.19	ns
CHCHD10	-1.94	ns	-2.30	2.15E-02	-0.37	ns
CKB	-1.62	ns	-1.84	4.57E-02	-0.22	ns
CNPPD1	-1.36	ns	-1.93	4.57E-02	-0.57	ns
COX8A	-0.71	ns	-1.73	4.32E-02	-1.01	ns
CT75	-4.33	ns	-4.38	4.57E-02	-0.05	ns
CWH43	-2.59	ns	-2.90	4.57E-02	-0.31	ns
DLG2	-4.31	ns	-4.89	2.41E-02	-0.58	ns
EDIL3	2.25	ns	3.63	7.96E-03	1.38	ns
ENSG00000188850	1.15	ns	1.44	3.61E-02	0.29	ns
ENSG00000290016	-0.92	ns	-1.20	4.21E-02	-0.28	ns
EPS15	0.55	ns	1.80	2.30E-02	1.26	ns
ESPN	0.51	ns	2.82	1.31E-02	2.31	ns
GPM6A	-2.57	ns	-3.18	3.45E-02	-0.61	ns
GSTK1	-1.53	ns	-2.39	3.87E-02	-0.87	ns
H2BC3	1.85	ns	2.30	4.73E-02	0.46	ns
HMGA2	2.64	ns	7.52	3.98E-02	4.88	ns
INF2	0.36	ns	1.64	4.32E-02	1.27	ns
IP6K3	-3.47	ns	-4.06	2.48E-02	-0.59	ns
ITPRIPL2	0.78	ns	1.29	2.52E-02	0.51	ns
KIAA0408	4.29	ns	6.42	2.15E-02	2.13	ns
KIF19	-4.81	ns	-5.73	1.86E-02	-0.92	ns
LINC02137	-1.35	ns	-1.57	3.45E-02	-0.23	ns
LINC02931	-0.70	ns	-0.84	3.45E-02	-0.14	ns
LINC02986	0.86	ns	1.86	3.91E-02	1.00	ns
lnc-CD8B2-4	-2.37	ns	-2.46	4.72E-02	-0.10	ns
LRRC37A11P	2.06	ns	2.87	4.58E-02	0.81	ns
MKLN1	2.11	ns	2.56	4.58E-02	0.45	ns
MT1F	-2.54	ns	-3.72	3.87E-02	-1.18	ns
MTX2	-0.51	ns	-0.76	4.32E-02	-0.25	ns
NDUFB11	-1.01	ns	-1.71	2.83E-02	-0.71	ns
NMRK2	-0.94	ns	-0.94	4.32E-02	0.00	ns
NUDT17	1.55	ns	2.12	4.58E-02	0.56	ns
PKHD1L1	-2.13	ns	-2.74	1.26E-02	-0.61	ns
PLXNC1	1.44	ns	2.43	4.19E-02	0.99	ns
PRPS1	-1.72	ns	-2.70	4.77E-02	-0.98	ns
PRR13P1	-0.46	ns	-1.31	3.50E-02	-0.85	ns
REV1	1.74	ns	2.06	2.15E-02	0.33	ns
SELENOV	-4.27	ns	-4.95	2.83E-02	-0.68	ns
SH2D6	-2.88	ns	-4.09	2.13E-02	-1.21	ns
STARD7	-0.63	ns	-1.49	3.45E-02	-0.86	ns
TBC1D19	1.74	ns	1.73	4.57E-02	-0.01	ns
THOC2	2.29	ns	2.99	3.48E-02	0.69	ns
TIA1	3.34	ns	4.15	3.19E-02	0.81	ns
TMEM11-DT	-1.41	ns	-1.45	4.58E-02	-0.04	ns
TMEM87A	2.58	ns	3.60	4.77E-02	1.02	ns
TMX2P1	-1.06	ns	-1.75	4.77E-02	-0.68	ns
VDAC3	-0.53	ns	-1.04	3.19E-02	-0.51	ns
YAF2	2.19	ns	3.37	3.44E-02	1.18	ns
YIF1A	-1.48	ns	-1.99	3.52E-02	-0.51	ns
YIPF1	-0.87	ns	-1.42	3.50E-02	-0.55	ns
ZNF432	1.51	ns	1.97	2.52E-02	0.46	ns

**Table 7:** Unique differentially expressed genes in RAI-R (highlighting genes specifically dysregulated in RAI-R cases). Log2FC: log2 fold change; AdjPval: adjusted p-value; ns: not significant; RAI-A: radioactive iodine-avid; RAI-R: radioactive iodine-refractory.

Gene symbol/Ensembl ID	RAI-A vs normal		RAI-R vs normal		RAI-R vs RAI-A	
	Log2FC	AdjPval	Log2FC	AdjPval	Log2FC	AdjPval
LINC02986	0.86	ns	1.86	3.91E-02	1.00	ns
LINC02137	-1.35	ns	-1.57	3.45E-02	-0.23	ns
ANTXR2	0.87	ns	1.44	6.39E-03	0.57	ns
SH2D6	-2.88	ns	-4.09	2.13E-02	-1.21	ns
lnc-CD8B2-4	-2.37	ns	-2.46	4.72E-02	-0.10	ns
CHCHD10	-1.94	ns	-2.30	2.15E-02	-0.37	ns
PRPS1	-1.72	ns	-2.70	4.77E-02	-0.98	ns
ENSG00000290016	-0.92	ns	-1.20	4.21E-02	-0.28	ns
COX8A	-0.71	ns	-1.73	4.32E-02	-1.01	ns
LINC02931	-0.70	ns	-0.84	3.45E-02	-0.14	ns
KIF19	-4.81	ns	-5.73	1.86E-02	-0.92	ns
CT75	-4.33	ns	-4.38	4.57E-02	-0.05	ns
DLG2	-4.31	ns	-4.89	2.41E-02	-0.58	ns
SELENOV	-4.27	ns	-4.95	2.83E-02	-0.68	ns
KIAA0408	4.29	ns	6.42	2.15E-02	2.13	ns
CA4	-3.74	ns	-3.93	4.77E-02	-0.19	ns
IP6K3	-3.47	ns	-4.06	2.48E-02	-0.59	ns
TIA1	3.34	ns	4.15	3.19E-02	0.81	ns
HMGA2	2.64	ns	7.52	3.98E-02	4.88	ns
CWH43	-2.59	ns	-2.90	4.57E-02	-0.31	ns
GPM6A	-2.57	ns	-3.18	3.45E-02	-0.61	ns
TMEM87A	2.58	ns	3.60	4.77E-02	1.02	ns
MT1F	-2.54	ns	-3.72	3.87E-02	-1.18	ns
ASPG	-2.36	ns	-3.12	1.87E-02	-0.77	ns
THOC2	2.29	ns	2.99	3.48E-02	0.69	ns
EDIL3	2.25	ns	3.63	7.96E-03	1.38	ns
C9orf92	-2.20	ns	-2.82	1.42E-02	-0.63	ns
YAF2	2.19	ns	3.37	3.44E-02	1.18	ns
PKHD1L1	-2.13	ns	-2.74	1.26E-02	-0.61	ns
MKLN1	2.11	ns	2.56	4.58E-02	0.45	ns
LRRC37A11P	2.06	ns	2.87	4.58E-02	0.81	ns
H2BC3	1.85	ns	2.30	4.73E-02	0.46	ns
TBC1D19	1.74	ns	1.73	4.57E-02	-0.01	ns
REV1	1.74	ns	2.06	2.15E-02	0.33	ns
CKB	-1.62	ns	-1.84	4.57E-02	-0.22	ns
NUDT17	1.55	ns	2.12	4.58E-02	0.56	ns
GSTK1	-1.53	ns	-2.39	3.87E-02	-0.87	ns
ZNF432	1.51	ns	1.97	2.52E-02	0.46	ns
ATP6AP1L	1.50	ns	1.63	4.58E-02	0.12	ns
YIF1A	-1.48	ns	-1.99	3.52E-02	-0.51	ns
PLXNC1	1.44	ns	2.43	4.19E-02	0.99	ns
TMEM11-DT	-1.41	ns	-1.45	4.58E-02	-0.04	ns
CNPPD1	-1.36	ns	-1.93	4.57E-02	-0.57	ns
ENSG00000188850	1.15	ns	1.44	3.61E-02	0.29	ns
TMX2P1	-1.06	ns	-1.75	4.77E-02	-0.68	ns
NDUFB11	-1.01	ns	-1.71	2.83E-02	-0.71	ns
NMRK2	-0.94	ns	-0.94	4.32E-02	0.00	ns
YIPF1	-0.87	ns	-1.42	3.50E-02	-0.55	ns
ITPRIPL2	0.78	ns	1.29	2.52E-02	0.51	ns
ATP5MC3	-0.77	ns	-1.32	4.57E-02	-0.54	ns
STARD7	-0.63	ns	-1.49	3.45E-02	-0.86	ns
EPS15	0.55	ns	1.80	2.30E-02	1.26	ns
VDAC3	-0.53	ns	-1.04	3.19E-02	-0.51	ns
MTX2	-0.51	ns	-0.76	4.32E-02	-0.25	ns
ESPN	0.51	ns	2.82	1.31E-02	2.31	ns
PRR13P1	-0.46	ns	-1.31	3.50E-02	-0.85	ns
INF2	0.36	ns	1.64	4.32E-02	1.27	ns

Differential expression of thyroid hormone-related genes in PTC subtypes

Several key genes involved in thyroid hormone synthesis and metabolism were differentially expressed in both RAI-A and RAI-R tissues compared to normal thyroid tissues (Table 6). Notably, TPO, DIO1, and SLC26A4 were consistently downregulated in both cancer subtypes.

TPO, a key enzyme in thyroid hormone biosynthesis, was significantly downregulated in RAI-A (log2FC = -15.84, AdjPval = 2.63E-03) and RAI-R (log2FC = -13.62, AdjPval = 5.87E-03), suggesting impaired thyroid hormone production in both subtypes. Although TPO expression was slightly higher in RAI-R than in RAI-A (log2FC = 2.22, AdjPval = 0.99), this difference was not statistically significant and may indicate residual differentiation capacity prior to the onset of refractoriness. Within the RAI-A group, RAIA3 and RAIA5 exhibited the lowest TPO expression values (12.99 and 11.09, respectively), contributing to intra-group heterogeneity.

TG, which encodes Tg, showed distinct variability across RAI-A samples. RAIA3 and RAIA5 had notably low expression (3.36 and 6.07), while the remaining samples demonstrated higher expression (ranging from 15.98 to 16.38). Overall, TG was downregulated in RAI-A compared to normal (log2FC = -4.69, AdjPval = 0.46), although the difference was not statistically significant. In contrast, TG expression in RAI-R was relatively consistent (16.10-16.84) and closely aligned with normal thyroid tissue (16.07-16.43). Compared to RAI-A, TG expression was higher in RAI-R (log2FC = 4.81, AdjPval = 0.99) and nearly unchanged relative to normal (log2FC = 0.12, AdjPval = 0.99), suggesting better preservation of thyroid differentiation.

TSHR, which encodes the receptor for TSH, was also downregulated in both subtypes, RAI-A (log2FC = -2.13, AdjPval = 0.44) and RAI-R (log2FC = -0.85, AdjPval = 0.78), although neither reached statistical significance. TSHR expression was slightly higher in RAI-R versus RAI-A (log2FC = 1.27, AdjPval = 0.99). Within the RAI-A group, RAIA3 (2.77) and RAIA5 (2.90) again showed the lowest TSHR expression, mirroring the pattern observed with TPO and TG.

DIO1 and DIO2, which are involved in the conversion of thyroid hormones, were downregulated in both PTC subtypes. DIO1 was significantly downregulated in RAI-A (log2FC = -3.39, AdjPval = 9.18E-05) and RAI-R (log2FC = -3.37, AdjPval = 1.01E-04), with no significant difference between groups (log2FC = 0.01, AdjPval = 0.99). DIO2 expression was also lower in RAI-A (log2FC = -4.46, AdjPval = 0.35) and RAI-R (log2FC = -3.89, AdjPval = 0.41), although these changes were not statistically significant.

Finally, iodide transporter genes SLC26A4 and SLC5A5 were downregulated in both RAI-A and RAI-R compared to normal thyroid. SLC26A4 showed similar expression levels in both groups (RAI-A: log2FC = -3.56, AdjPval = 0.34; RAI-R: log2FC = -3.44, AdjPval = 0.33). In contrast, SLC5A5, which encodes NIS, showed minimal changes (RAI-A: log2FC = -0.46, AdjPval = 0.74; RAI-R: log2FC = -0.24, AdjPval = 0.89), indicating relatively stable gene expression across subtypes despite impaired iodide uptake in RAI-R.

Pathway enrichment analysis

GSEA using the KEGG pathway database identified several significantly altered biological pathways in both RAI-A and RAI-R tissues when compared to normal thyroid tissues. In the RAI-R versus normal comparison, the downregulated pathways included drug metabolism (NES = -0.64, AdjPval = 5.00E-03), carbon metabolism (NES = -0.51, AdjPval = 5.00E-03), and tyrosine metabolism (NES = -0.65, AdjPval = 1.00E-02). Notably, the thyroid hormone synthesis pathway was significantly downregulated (NES = -0.54, AdjPval = 1.30E-02), suggesting an impairment in thyroid hormone production and loss of thyroid-specific function in RAI-R. In contrast, upregulated pathways in RAI-R included those related to immune responses and viral infections, such as herpes simplex virus 1 infection (NES = 0.44, AdjPval = 4.40E-05) and transcriptional misregulation in cancer (NES = 0.45, AdjPval = 4.90E-03), reflecting enhanced or evasion immune signaling and transcriptional dysregulation in RAI-R tumors.

In the RAI-A versus normal comparison, a similar downregulation was observed in thyroid hormone synthesis (NES = -0.62, AdjPval = 4.00E-04), drug metabolism-cytochrome P450 (NES = -0.61, AdjPval = 1.40E-02), and tyrosine metabolism (NES = -0.64, AdjPval = 2.50E-02), indicating shared disruptions in metabolic and differentiation-related pathways between both PTC subtypes. However, RAI-A displayed distinct upregulated pathways including ribosome biogenesis (NES = 0.67, AdjPval = 6.20E-10), systemic lupus erythematosus (NES = 0.58, AdjPval = 1.20E-05), and cell cycle-related processes (NES = 0.50, AdjPval = 1.90E-03), indicating enhanced proliferative activity in RAI-A.

Thyroid hormone synthesis pathway in RAI-A and RAI-R

**Figure 2:**
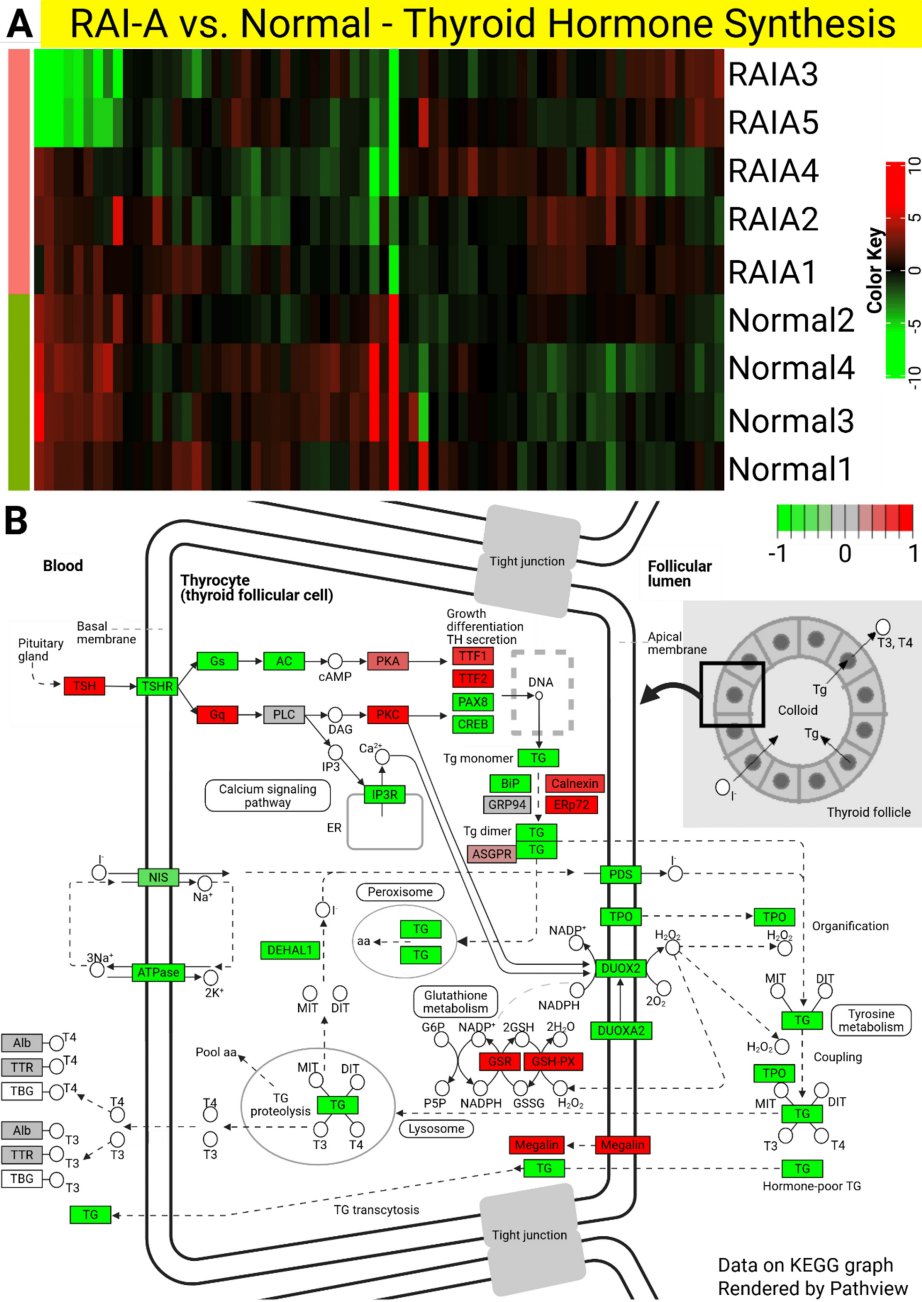
Heatmaps and pathway analysis of differentially expressed genes (DEGs) involved in thyroid hormone synthesis in RAI-A. (A) Heatmap representing the expression patterns of genes involved in thyroid hormone synthesis in RAI-A compared with normal thyroid tissues. Each row represents a gene, and each column represents a sample, with green indicating downregulation and red indicating upregulation. A clear distinction in expression patterns was observed between RAI-A samples and normal tissues. (B) KEGG pathway analysis illustrating the involvement of DEGs in the thyroid hormone synthesis pathway in RAI-A. Upregulated genes are shown in red, and downregulated genes are shown in green, indicating significant changes in the pathway regulation in RAI-A samples. RAI-A: radioactive iodine-avid; KEGG: Kyoto Encyclopedia of Genes and Genomes. The figure was originally created by the authors and developed specifically for this manuscript.

**Figure 3:**
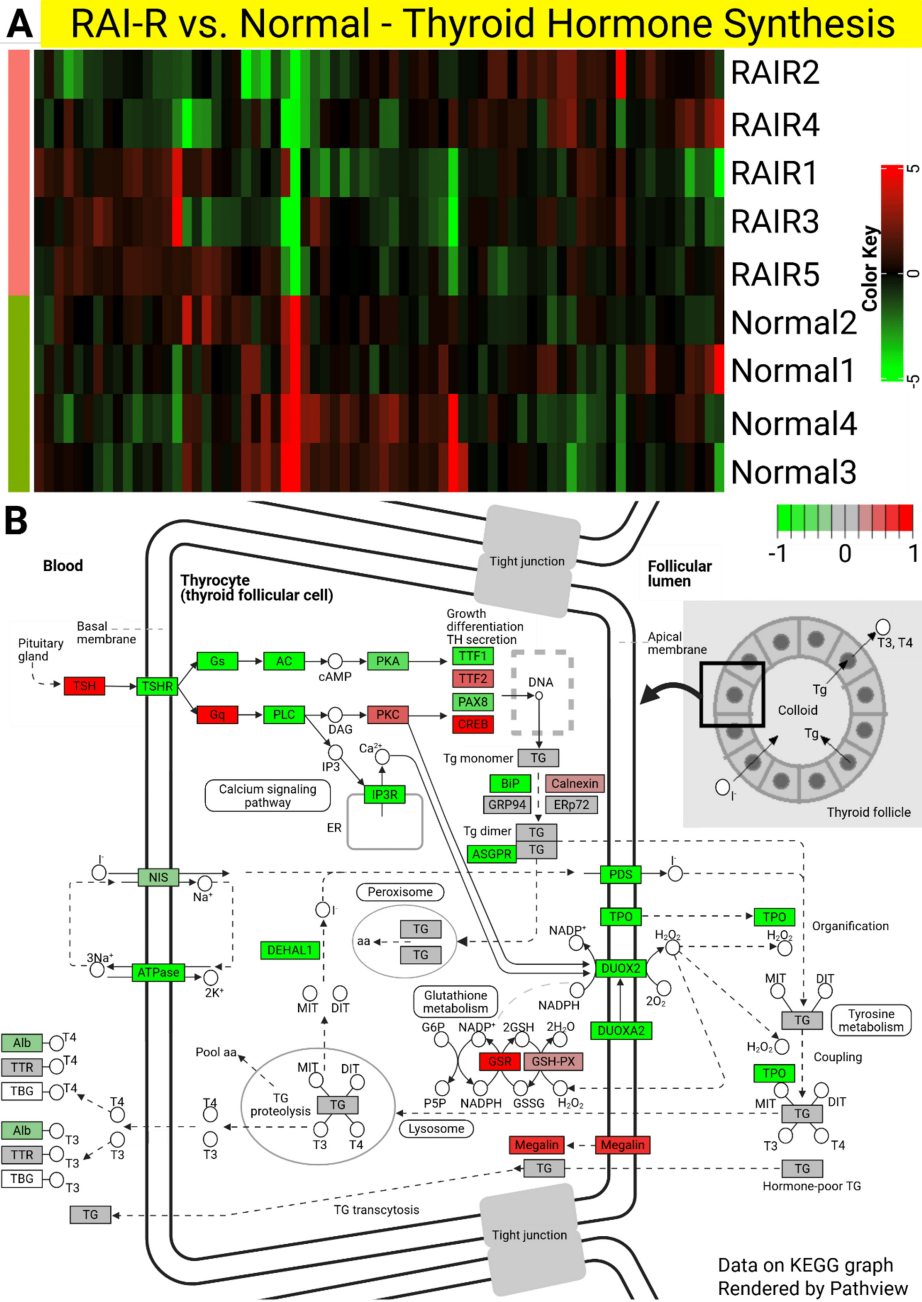
Heatmaps and pathway analysis of differentially expressed genes (DEGs) involved in thyroid hormone synthesis in RAI-R. (A) Heatmap representing the expression patterns of genes involved in thyroid hormone synthesis in RAI-R compared with normal thyroid tissues. Each row represents a gene, and each column represents a sample, with green indicating downregulation and red indicating upregulation. A clear distinction in expression patterns was observed between normal tissues and RAI-R samples. (B) KEGG pathway analysis illustrating the involvement of DEGs in the thyroid hormone synthesis pathway in RAI-R. Upregulated genes are shown in red, and downregulated genes are shown in green, indicating significant changes in the pathway regulation in RAI-R samples. RAI-R: radioactive iodine-refractory; KEGG: Kyoto Encyclopedia of Genes and Genomes. The figure was originally created by the authors and developed specifically for this manuscript.

Focusing on the thyroid hormone synthesis pathway, which plays a crucial role in thyroid function, we observed significant downregulation in both RAI-A and RAI-R tissues compared to normal tissues (Figures 2, 3). The heatmaps (Figures 2A, 3A) show clear DEGs involved in this pathway across the samples, with RAI-A and RAI-R displaying distinct gene expression patterns compared with normal thyroid tissues. The pathway maps (Figures 2B, 3B) further visualized the involvement of these DEGs, with several key components downregulated in both RAI-A and RAI-R.

Downregulation of thyroid hormone synthesis-related genes suggests a disruption in hormone production, which may contribute to the clinical phenotypes observed in these PTC subtypes. In RAI-A, the reduced expression of genes such as DIO1 and TG correlates with RAI-A, whereas in RAI-R, additional dysregulation of genes involved in hormone synthesis may be associated with RAI-R. These findings highlight the importance of the thyroid hormone synthesis pathway in the pathophysiology of PTC and highlight the molecular differences between RAI-A and RAI-R.

## Discussion

This study aimed to identify clinicopathological and molecular features associated with RAI-R PTC. Our findings reveal distinct differences between RAI-R and RAI-A cases, particularly in age, staging, lymph node involvement, histology, and gene expression, underscoring the multifactorial nature of RAI-R.

RAI-R patients were generally older at diagnosis, consistent with previous studies reporting age 55 years or older as a significant risk factor for RAI resistance [5,23]. This may reflect biological shifts associated with aging, such as diminished immune surveillance, altered tumor microenvironment, and reduced NIS activity. However, age alone is insufficient for predicting refractoriness and must be evaluated alongside molecular and pathological features.

Although gender was not statistically associated with RAI response, all male patients in our cohort were RAI-R. Some studies suggest that male sex may be linked to aggressive disease and reduced iodine uptake [24,25], though others report no clear association [26,27]. The apparent trend in our cohort warrants further investigation in larger, sex-stratified studies.

Tumor size is frequently regarded as a prognostic indicator, but our results showed that large tumors measuring 4 cm or more were also found in RAI-A patients, indicating that size alone does not predict iodine avidity. Although larger tumors are often linked to dedifferentiation and reduced NIS expression [12], other studies found no consistent association [6,27]. These findings suggest that tumor size, while clinically important, should be considered alongside molecular and histological features when assessing RAI-R.

Advanced staging was more common in RAI-R cases, consistent with previous studies linking higher stages to poor RAI response [4,13,28]. However, this association often weakens in multivariate analyses, indicating that iodine avidity may be governed more by molecular and histological factors than by anatomical extent alone. Thus, staging remains clinically useful but should be interpreted in conjunction with tumor biology for accurate prognostication.

LNM, especially lateral involvement and high LNM ratio, was more frequent in RAI-R patients. These findings are consistent with prior studies linking LNM burden to poor RAI response [4,25,29]. However, other studies found no significant association [28], suggesting that LNM alone lacks predictive specificity. Rather than serving as an independent marker, extensive LNM may reflect broader tumor aggressiveness, underscoring the need to evaluate nodal status alongside molecular and histological features.

Histologically, the classic PTC variant predominated in both groups. However, one RAI-R patient harbored the tall cell variant, known for its association with aggressive behavior and poor iodine uptake [5,27,30]. Nilsson et al. [6,7] further confirmed that tall cell, hobnail, and oxyphilic variants exhibit significantly reduced RAI uptake. These findings underscore the importance of histological subtyping in prognostication and treatment planning, as unfavorable variants are more likely to exhibit resistance to RAI therapy.

At the molecular level, microarray analysis revealed downregulation of thyroid hormone synthesis genes (TPO, TG, TSHR, and SLC5A5) in both RAI-A and RAI-R tumors relative to normal thyroid tissue, indicating dedifferentiation. This aligns with previous studies showing that loss of thyroid-specific gene expression contributes to RAI resistance [13,31].

TPO, essential for organification of iodide, was markedly downregulated in tumor tissues. Though no statistically significant difference was found between the RAI-R and RAI-A groups, two RAI-A samples (RAIA3 and RAIA5) showed notably low expression. This may suggest subclinical dedifferentiation or transitional states, reflecting early loss of iodine-handling capacity. Previous studies have shown that tumors can initially respond to RAI before progressing to refractoriness, particularly those with BRAFV600E or TERT promoter mutations [7,9].

TSHR expression was slightly downregulated in both RAI-A and RAI-R tumors, but without statistical significance. Nilsson et al. [7] found only a weak correlation between TSHR levels and iodine avidity, and Li et al. [32] demonstrated that TSHR remains consistently expressed in metastatic and RAI-R lesions. While TSHR may have limited prognostic utility, its stability suggests potential therapeutic applications, such as TSHR-directed CAR-T cell therapies.

SLC5A5, encoding NIS, was slightly downregulated in both RAI-A and RAI-R tumors compared to normal tissue, though not statistically significant. Marginally higher expression in RAI-R suggests possible subclonal heterogeneity or transitional changes in NIS function [33-35]. Importantly, NIS functionality depends on proper membranous localization, which was not evaluated in this study. A previous study has shown that cytoplasmic NIS lacks predictive value unless correctly trafficked to the membrane [7]. However, strong correlations between membranous NIS and iodine uptake [11,13] support SLC5A5's potential as a therapeutic redifferentiation target.

TG expression showed an unexpected trend: lower in RAI-A compared to RAI-R tumors, although not statistically significant. In differentiated PTC, Tg is typically expressed at high levels and is associated with preserved thyroid function and favorable prognosis [6,36]. The observed variation within the RAI-A group, including low TG levels in RAIA3 and RAIA5, may indicate early dedifferentiation. Moreover, reduced TG expression in metastatic lesions correlates with higher Ki-67 indices and shorter recurrence-free survival [37], supporting its role as a prognostic marker. This intra-group heterogeneity, particularly in TG expression among RAI-A samples, may reflect subclonal evolution or early dedifferentiation states, complicating group-level interpretations.

Importantly, all RAI-R patients in this study demonstrated initial RAI uptake on early scans but later developed refractoriness. Although longitudinal data were limited, this pattern supports the hypothesis that RAI resistance can be acquired rather than intrinsic. This emphasizes the importance of longitudinal molecular monitoring to detect emerging resistance. Mechanisms such as subclonal evolution, epigenetic silencing, and altered NIS trafficking may contribute to this progression [33-35]. Therefore, iodine avidity should not be considered a static trait but one shaped by dynamic tumor evolution.

This study offers valuable insights into transcriptomic differences between RAI-A and RAI-R PTC in Malaysian patients, supported by a well-controlled design and comprehensive analysis. Key strengths include clearly defined patient groups, standardized microarray processing, and robust enrichment analysis highlighting thyroid hormone synthesis and immune-related pathways. However, the study has several limitations. The small sample size (n = 5 per group) reduces statistical power and limits generalizability. While efforts were made to control technical variation, sample heterogeneity, especially the variable TG expression in RAI-A samples, may have influenced the results. Functional validation at the mRNA or protein level (e.g., qPCR and IHC) was not performed, and key protein-level metrics such as membranous NIS localization were not assessed. Moreover, some gene expression trends (e.g., upregulation of TSHR, TPO, and SLC5A5 in RAI-R relative to RAI-A) diverged from existing literature, which may reflect population-specific biology or technical variation. Although batch correction was not applied, balanced sample distribution across microarray chips reduced the likelihood of batch-related effects. These limitations highlight the need for follow-up studies involving larger, independent cohorts and functional assays to validate and extend these preliminary findings.

## Conclusions

Our findings highlight the multifactorial nature of RAI-R, shaped by clinical, pathological, and molecular interactions. RAI-R cases were more frequently associated with older age, advanced stage, LNM, and tall cell histology. Transcriptomic analysis revealed consistent yet heterogeneous downregulation of key thyroid differentiation genes in both RAI-A and RAI-R tumors, indicating impaired iodine-handling capacity. Notably, early signs of dedifferentiation were also observed in some RAI-A tumors, suggesting a potential continuum of radioiodine responsiveness. Additionally, altered immune signatures in RAI-R cases may contribute to therapeutic resistance. These findings offer preliminary molecular insights that may help identify biomarkers of RAI response and inform future therapeutic targets. Despite limitations such as small cohort size, lack of protein-level validation, and clinical heterogeneity, our study supports the integration of transcriptomic and clinicopathological data to improve the prediction of RAI response and guide personalized management in PTC. Importantly, these results serve as a foundation for hypothesis-driven validation in future mechanistic studies.
